# Analgesic efficacy of continuous femoral nerve block commenced prior to operative fixation of fractured neck of femur

**DOI:** 10.1186/2047-0525-1-4

**Published:** 2012-06-27

**Authors:** Szilard Szucs, Gabriella Iohom, Brian O’Donnell, Pavol Sajgalik, Ishtiaq Ahmad, Nazar Salah, George Shorten

**Affiliations:** 1 Department of Anaesthesia, Intensive Care and Pain Medicine, Cork University Hospital, Wilton, Cork, Ireland; 2 Department of Anaesthesia, Intensive Care and Pain Medicine, University College Cork, Wilton, Cork, Ireland

**Keywords:** Perioperative pain relief, Hip fractured neck of femur, Continuous femoral nerve block

## Abstract

**Background:**

Peripheral nerve blocks are effective in treating acute pain, thereby minimizing the requirement for opiate analgesics. Fractured neck of femur (FNF) is a common, painful injury. The provision of effective analgesia to this cohort is challenging but an important determinant of their functional outcome. We investigated the analgesic efficacy of continuous femoral nerve block (CFNB) in patients with FNF.

**Methods:**

Following institutional ethical approval and with informed consent, patients awaiting FNF surgery were randomly allocated to receive either standard opiate-based analgesia (Group 1) or a femoral perineural catheter (Group 2). Patients in Group 1 received parenteral morphine as required. Those in Group 2 received a CFNB comprising a bolus of local anaesthetic followed by a continuous infusion of 0.25% bupivacaine. For both Groups, rescue analgesia consisted of intramuscular morphine as required and all patients received paracetamol regularly. Pain was assessed using a visual analogue scale at rest and during passive movement (dynamic pain score) at 30 min following first analgesic intervention and six hourly thereafter for 72 hours. Patient satisfaction with the analgesic regimen received was recorded using verbal rating scores (0-10). The primary outcome measured was dynamic pain score from initial analgesic intervention to 72 hours later.

**Results:**

Of 27 recruited, 24 patients successfully completed the study protocol and underwent per protocol analysis. The intervals from recruitment to the study until surgery were similar in both groups [31.4(17.7) vs 27.5(14.2) h, P = 0.57]. The groups were similar in terms of baseline clinical characteristics. For patients in Group 2, pain scores at rest were less than those reported by patients in Group 1 [9.5(9.4) vs 31(28), P = 0.031]. Dynamic pain scores reported by patients in Group 2 were less at each time point from 30 min up to 54 hours [e.g at 6 h 30.7(23.4) vs 67.0(32.0), P = 0.004]. Cumulative morphine consumption over 72 h was less in Group 2. Patient satisfaction scores were greater in Group 2 [9.4(1.1) vs 7.6(1.8), P = 0.014].

**Conclusions:**

CFNB provides more effective perioperative analgesia than a standard opiate-based regimen for patients undergoing fixation of FNF. It is associated with lesser opiate use and greater patient satisfaction.

## Background

Fractured neck of femur (FNF) is a common, painful reason for hospital admission in elderly patients [1]. Pain management in the elderly can be challenging due to the presence of co-morbidities, altered pharmacokinetics and pharmacodynamics. Despite clinical guidelines favoring surgical repair of FNF within 24 hours of injury [2] patients may wait considerable periods of time for their turn in the operating room. In this context, preoperative pain is an important distressing factor.

The sensory innervation of the proximal femur and a variable portion of the intra-capsular neck of femur arise from the femoral nerve [3]. Femoral nerve block is effective in providing analgesia for femur factures, and has been previously described in FNF [4]. Perineural catheter placement permits the provision of continuous peripheral nerve block, thereby extending the duration of analgesia. Continuous femoral nerve block (CFNB) may therefore have a role in the provision of high quality analgesia in patients awaiting surgery for FNF. Such regional analgesia techniques may improve the quality of pain relief and potentially limit both opiate use and associated opiate-related side effects [5].

It is not known whether CFNB improves analgesic outcomes in elderly patients presenting acutely with FNF. We conducted a study to compare the analgesic efficacy of CFNB and conventional parenteral opiate analgesia in this patient group. Our hypothesis states that continuous femoral nerve block provides better peri-operative analgesia than standard parenteral opiate regimens in patients awaiting surgery to repair FNF.

## Methods

Ethical approval was granted by the Clinical Research Ethics Committee of the Cork Teaching Hospitals. Written, informed consent was obtained from all patients. Patients presenting via the emergency room of Cork University Hospital with fractured neck of femur, American Society of Anesthesiologists grades I to III and aged above 50 years, were invited to participate in the study. Exclusion criteria included patient refusal, the presence of more than one fracture; Mini-Mental Score <22 [6]; coagulation disorders; head injury; loss of consciousness; 10 mg or more morphine administration pre-hospital; acute intercurrent heart disease; allergy to bupivacaine, morphine or paracetamol; skin lesions/infection at block site; and renal dysfunction. Patients with evidence of systemic infections (clinically defined or elevated C-reactive protein levels, leucocytosis, or body temperature higher than 37.8°C) were also excluded.

On recruitment to the study, patients were randomized using a random number sequence and sealed envelopes. Those randomized to Group 1 received standard analgesia consisting of paracetamol 1 g po 6 hourly and parenteral morphine up to 0.1 mg/kg im 4 hourly as required. Patients in Group 2 received 10 ml of 2% lidocaine and 10 ml of 0.5% bupivacaine after repeated negative aspirations slowly over two to three minutes via a perineural femoral catheter followed by 0.25% bupivacaine infused at 4 ml per hour for 72 hours. They also received paracetamol 1 g po 6 hourly. Breakthrough pain in Group 2 was treated with intramuscular morphine as required.

Cyclizine 50 mg im 8 hourly as required was used to treat nausea and vomiting.

Anaesthesia for surgical repair FNF was provided using an intrathecal block. Fifteen minutes prior to positioning for spinal anaesthesia, a lidocaine bolus (10 ml 2% lidocaine) was administered through the catheter. On positioning for spinal anaesthesia (fractured limb dependent in view of using weight/height appropriate dose of hyperbaric bupivacaine), additional analgesia was provided at the discretion of the attending anaesthetist.

### Continuous femoral block technique

Having attached standard monitoring (non-invasive blood pressure, oxygen saturation and electrocardiography) and inserted a peripheral intravenous cannula, the femoral catheter was placed, in the emergency department, using nerve stimulation by the primary investigator (SS). The needle insertion point was first determined using predefined landmarks. A skin mark was placed one centimeter caudal to the inguinal ligament and one centimeter lateral to the point of maximal palpable pulsation of the femoral artery.

The skin of the anterior thigh was prepared aseptically and a sterile drape was placed. The skin was anaesthetised using a 25 G hypodermic needle and 1% lidocaine. The block needle (Contiplex, BBraun, Melsungen, Germany) was attached to a nerve stimulator set at 2 mA with 2 Hz pulse cycle and pulse duration of 0.1 ms. Appropriate needle position was determined by the presence of quadriceps contractions resulting in patellar movement at a current of 0.4 mA. On attaining this endpoint the needle was immobilized, and following negative aspiration 10 ml 2% lidocaine was injected. The Contiplex cannula was then advanced over the needle, the needle withdrawn and the catheter placed through the cannula 3 cm in cephalad direction. Finally, the cannula was removed and the catheter secured to the skin using an adhesive, transparent dressing. The patient received 10 ml 0.5% bupivacaine, following which a continuous infusion of 0.25% bupivacaine was commenced at 4 ml per hour, delivered via an elastomeric pump (Acemedical, AutoFuser, Seoul, South Korea).

### Primary outcome

The primary outcome measure was pain assessed using visual analogue score (VAS 0-100) on passive movement (30 degree flexion) of the injured limb from initial analgesic intervention until 72 hours thereafter.

### Secondary outcomes

Visual analogue scores for pain were measured at rest and passive movement at recruitment, 30 minutes after recruitment and 6 hourly for the next 72 hours. Passive movement was defined as 30 degree flexion of thigh. Pain on positioning for spinal anaesthesia was also recorded (Verbal Rating Score, VRS 0-10). Satisfaction with the analgesic regimen received was measured at the end of the assessment period using a VRS (0-10).

Patients were evaluated for i. Nausea/Vomiting, ii. Pruritus and iii. Excessive sedation (4 on a observational scale 1-4) immediately after initial analgesic intervention and six hourly thereafter for 72 hours.

Adverse events were recorded by the attending anaesthetist on a dedicated data sheet.

### Statistical analysis

Our study was powered to detect a 50% reduction in VAS pain score six hours after recruitment. With alpha error rate of 0.05 and power of 0.80, it was estimated that 24 patients would be required. Assuming 15% exclusion rate, we planned to recruit 27 patients. Statistical analysis was performed using EpiInfo™ 2002 (Centers for Disease Control and Prevention) statistics software. Quantitative data were analyzed using ANOVA or Fisher Exact test. Categorical data were examined by Kruskal-Wallis test.

## Results

With initial ethics approval and having obtained written informed consent 27 (of 57 approached) patients were recruited to the study. Three patients were subsequently excluded leaving 24 patients for final analysis (12 patients in Group 1; 12 patients in Group 2). Patients were excluded for the following reasons: (1) elastomeric pump failure resulting in the local anaesthetic administered over less than 54 hours instead of 72 hours, (2) patient confusion with subsequent pump disconnection after 12 hours, (3) late diagnosis of a complicating acetabular fracture.

The two groups were similar in terms of baseline characteristics (Table 1), time to surgery and VRS at positioning for spinal anaesthesia. Overall satisfaction recorded at the 72 hour time point was greater in group 2 (Table 2).

**Table 1:** Baseline characteristics

	**Group 1**	**Group 2**
	**N=12**	**N=12**
**Age (year), mean (SD)**	**80.2(5.1)**	**76.0(13.7)**
**Female/Male**	**10/2**	**6/6**
**FNF side left:right**	**4/8**	**8/4**

**Table 2:** Time to surgery, pain at positioning before spinal and satisfaction scores

	**Group 1**	**Group 2**
	**N=12**	**N=12**
**Time to surgery, mean(SD)**	**27.0(3.6)**	**31.45(17.9)**
**VRS before spinal anaesthesia, mean(SD)**	**6.44(2.7)**	**3.72(3.2)**
**Overall satisfaction VRS, mean(SD)**	**7.6(1.8)**	**9.4(1.1), P=0.01**

All femoral nerve blocks including insertion and securing the femoral nerve catheter were completed in less than 15 minutes.

**Figure 1:**
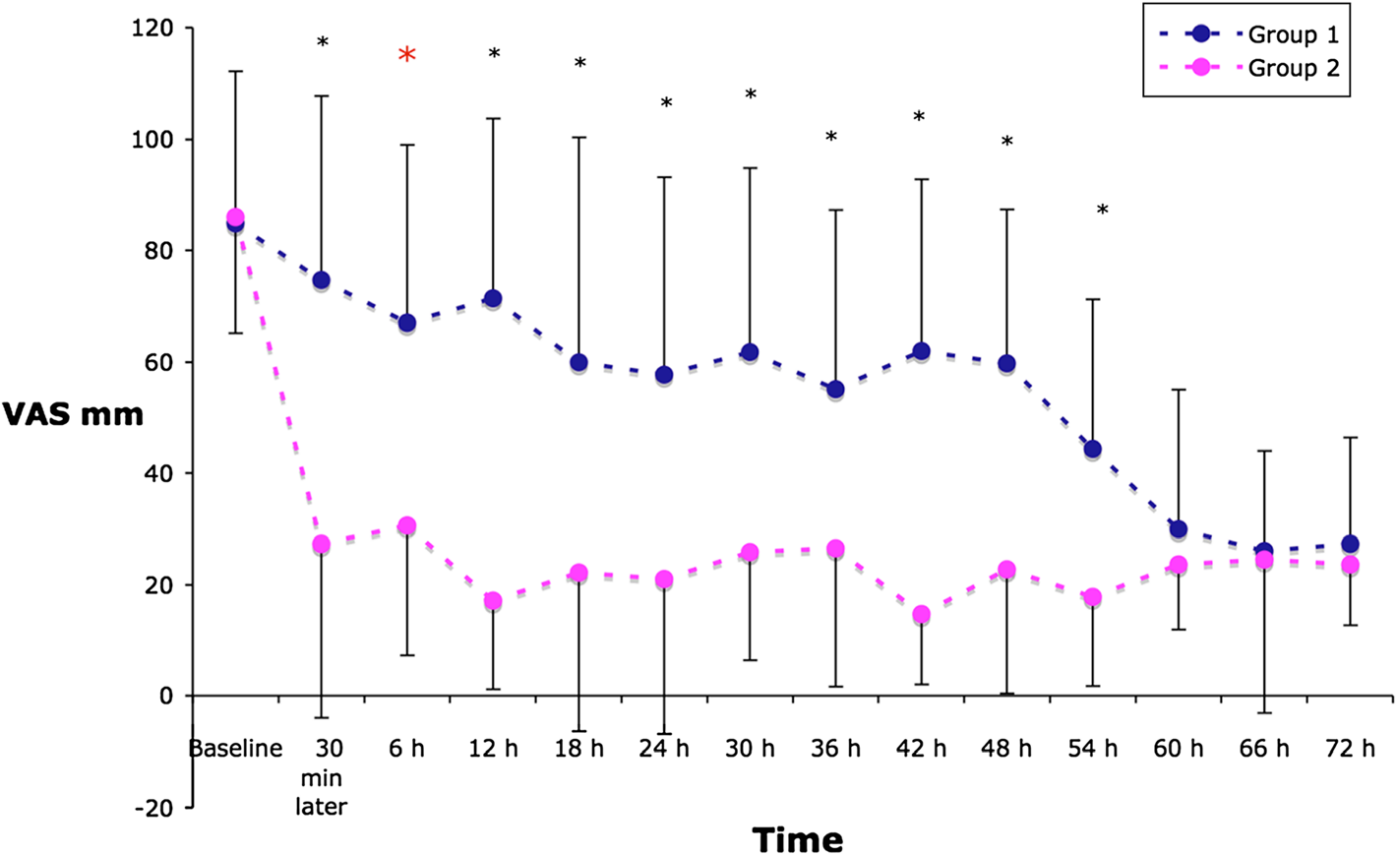
VAS pain scores at passive movement.

**Figure 2:**
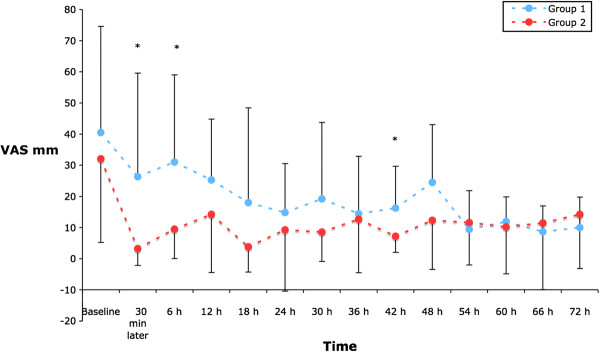
VAS pain scores at rest.

**Figure 3:**
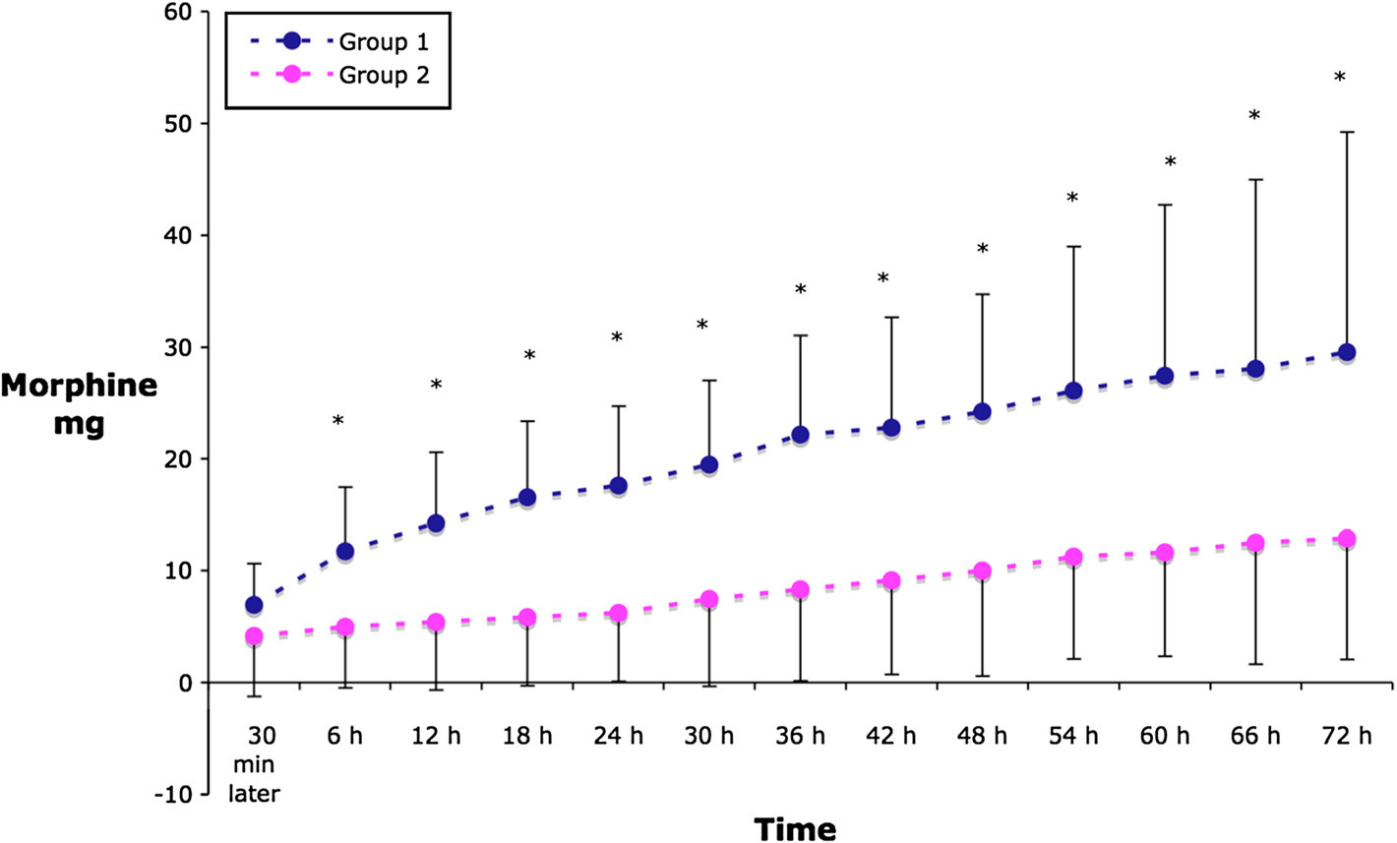
Cumulative morphine consumption.

Patients in Group 1 reported greater pain scores (VAS) on passive movement at 6 hours compared to Group 1 [30.7(23.4) vs 67.0(32) mm, p = 0.004]. Pain measured during passive movement was less in Group 2 at each time point up to 54 hours (Figure 1). Similarly, pain measured at rest was consistently less in Group 2 at all time points, reaching the statistical significance level 30 minutes, six and 42 hours after recruitment (Figure 2). Cumulative morphine consumption was lower in Group 2 at each time point except at 30 minutes after recruitment (Figure 3).

**Figure 4:**
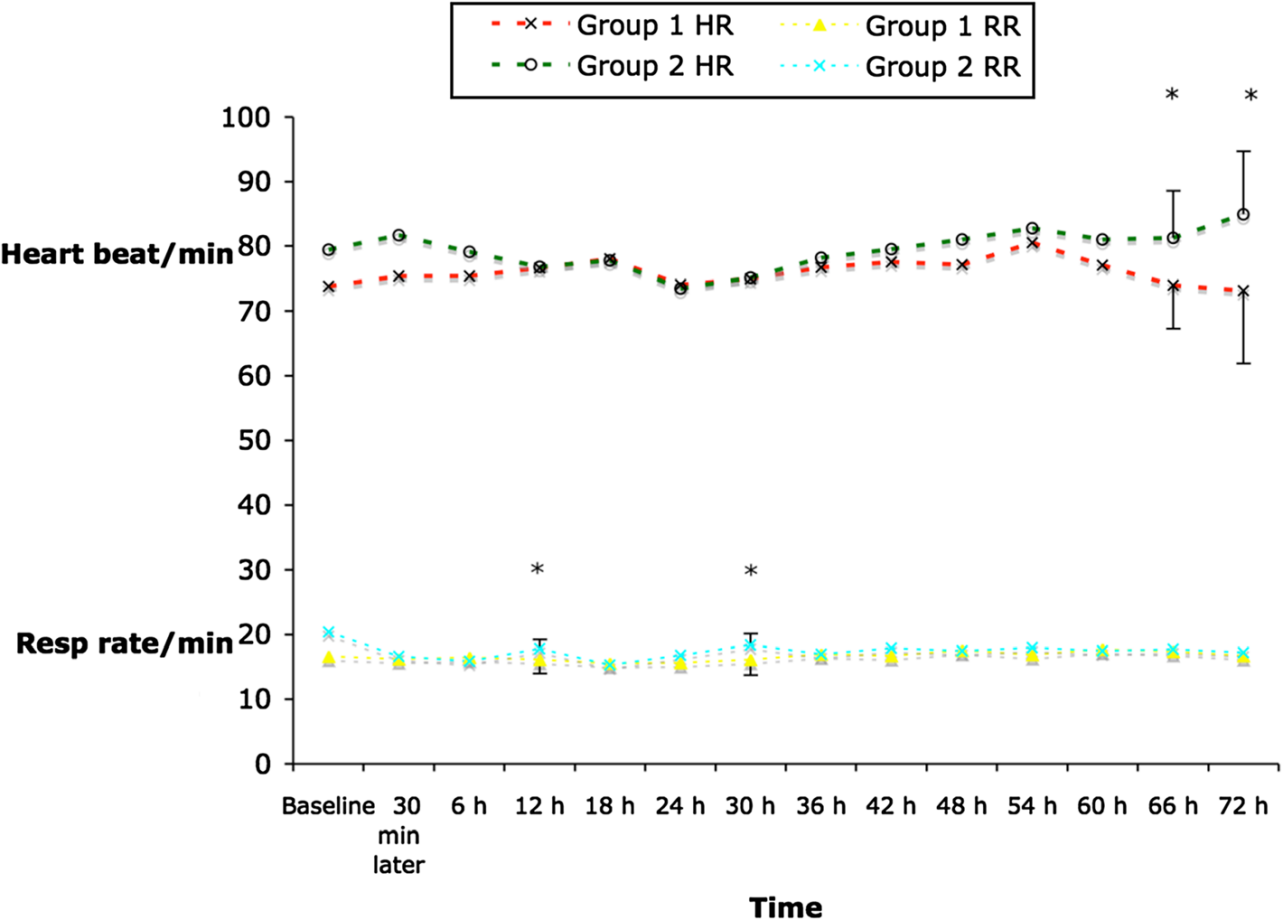
Haemodynamical parameters.

Hemodynamic parameters were not different between groups perioperatively up to 66 hours post recruitment. At 66 and 72 hours patients in Group 2 had higher heart rate compared to those in Group 1, i.e. 81.71(7.29) vs 74.09(6.70) bpm, P = 0.03 and 84.88(9.84) vs 73.27 (11.03) bpm, P = 0.02, respectively (Figure 4). Respiratory rate was higher in Group 1 compared to Group 2 at 12 h [(17.81 (1.40) vs 16.16 (2.16) per minute, P = 0.04] and 30 h post recruitment [18.36(1.74) vs 16.18(2.04) per minute, P = 0.01] (Figure 4).

The intervals from recruitment to the study until surgery is similar to intervals reported in a previous study [7] and were similar in both groups [27.1(13.6) h vs. 31.5(17.9), P = 0.25]. Pharmacological agents used for sedation and analgesia for positioning of patients in Group 1 were fentanyl (1 instance), fentanyl plus midazolam (3 patients), propofol plus fentanyl (2 patients). No medication was administered for this purpose to patients in Group 2.

The incidence of nausea/vomiting [3/12 (25%) vs. 4/12 (33%) in Group 1 and 2 respectively], pruritus [2/12 (16.6%) vs. 1/12 (8.3%) in Group 1. and 2. respectively] were similar in the two groups (P<0.05). The incidence of excessive sedation was also similar in the two groups [1/12 (8.3%) vs. 1/12 (8.3%) in both group].

A trend towards lesser pain sensation (VRS) was identified in Group 2 vs Group 1 at positioning for spinal anaesthesia [3.7(3.2) vs 5.4(2.7), P = 0.10], although this did not reach statistical significance.

Scores for patients satisfaction with analgesia overall were greater in Group 2 [9.4(1.1) vs 7.6(1.8), P = 0.014].

## Discussion

The most important finding of our study was that continuous femoral nerve blockade offered superior analgesia compared to systemic opioids in the period around operative fixation of fractured neck of femur. In addition, CFNB it was associated with greater patient satisfaction.

Best practice review of the care of patients with fractured neck of femur included a continuous femoral nerve block as analgesia in the Emergency Department [8], however this is not common practice. When performed at all, usually a single shot femoral nerve block is administered by physicians in the emergency department [9] or in the pre-hospital setting [10].

Our study demonstrated feasibility of continuous femoral nerve block in this clinical context. The femoral perineural catheter was successfully placed in each of the 15 patients randomized to Group 2. The true economic input of the use of perineural catheters and elastomeric pumps requires further evaluation.

Opioid consumption was not eliminated by the presence of a perineural catheter. This may account for the presence of morphine associated side effects in this group. A logical explanation for this is the sciatic contribution to the innervation to the femur and that of the lateral cutaneous nerve of the thigh to the surgical incision in the postoperative period. Our chosen continuous infusion regime, while limiting local anaesthetic dose and potential toxicity, may have decreased the spread of local anaesthetic towards the lateral cutaneous nerve of the thigh.

In our study, the average intervals between initial analgesic intervention and surgery were 27.1 and 31.5 hours (Groups 1 and 2 respectively). Therefore the first bolus of 10 mls of bupivavacaine probably had minimal effect at the time of surgery. We believe that one of the benefits of the combined bolus + continuous infusion is that it is suitable in a setting in which the duration of the need for potent analgesia is variable and unpredictable (such as for patients with FNF). Cuvillon et al [11] have demonstrated that the duration of a single bolus of bupivacaine 0.5% 20 mL for FNB is 22 h (range 15-32). Thus the analgesic benefits (in the 72 hour study interval defined for this investigation) of the CFNB technique were of greater importance preoperatively.

There are several limitations to this study. For ethical and economic reasons, it was not possible to use a double-blinded methodology. The authors considered it to be ethically unacceptable to insert a placebo femoral nerve catheter for blinding purposes only. At our institution, the standard dressing employed for securing a femoral nerve catheter comprises a transparent adhesive layer (usually Tegaderm^TM^ Film, 3 M). This made it unfeasible to apply a “dummy” catheter to the groin. A patient controlled analgesia (PCA) pump would have allowed a more precise measurement of parenteral opioid consumption. Analgesia for positioning prior to spinal anaesthesia was not standardized, and may account for the observed results. Outcomes such as time to mobilization, postoperative respiratory or cardiovascular morbidities and time to achieve discharged criteria were not assessed. One cohort of patients, the confused elderly, which might be expected to benefit most from this intervention were not studied for ethical reasons (difficulty ensuring that consent was informed). The interval from initiating analgesic management until surgery were similar in the two Groups. As we arbitrarily selected a cut-off time of 72 hours for the continuous perineural blockade, our results contain both pre- and postoperative parameters. We did not specifically address whether any benefits associated with the catheter occurred pre- or postoperatively.

Although ultrasound guidance was not used in this study, we believe that it would enhance the benefits of the CFNB technique. Specifically it may minimize the patient discomfort associated with use of peripheral nerve stimulation during the nerve block procedure and, in expert hands, may decrease the likelihood of block failure or nerve injury.

Our study reflects other available evidence substantiating the use of continuous peripheral nerve block analgesia in FNF [12]. Whether this has an impact on early mobilization or long term rehabilitation requires further research.

## Conclusions

We conclude that, compared with a systemic opiate based regimen, continuous femoral nerve blockade provides superior perioperative analgesia for patients undergoing operative fixation of fractured neck of femur.

## Competing interests

The authors declare that they have no competing interests.

## Authors’ contributions

GI: participated in the design of the study, coordination of data collection and in manuscript preparation. BO’D: participated in the design of the study and manuscript preparation. PS: participated in the data collection and in statistical analysis. IA: participated in the data collection. NS: participated in the data collection. GS: participated in the design of the study, coordination of data collection and manuscript preparation. All authors read and approved the final manuscript.
